# Reasons for faculty departures from an academic medical center: a survey and comparison across faculty lines

**DOI:** 10.1186/s12909-016-0830-y

**Published:** 2017-01-10

**Authors:** Sabine C. Girod, Magali Fassiotto, Roseanne Menorca, Henry Etzkowitz, Sherry M Wren

**Affiliations:** 1 Department of Surgery, Stanford University School of Medicine, 94305 Stanford, CA USA; 2 Office of Faculty Development and Diversity, Stanford University School of Medicine, 94305 Stanford, CA USA; 3 Science and Technology Society Program, Stanford University and International Triple Helix Institute, Palo Alto, CA USA

## Abstract

**Background:**

Faculty departure can present significant intellectual costs to an institution. The authors sought to identify the reasons for clinical and non-clinical faculty departures at one academic medical center (AMC).

**Method:**

In May and June 2010, the authors surveyed 137 faculty members who left a west coast School of Medicine (SOM) between 1999 and 2009. In May and June 2015, the same survey was sent to 40 faculty members who left the SOM between 2010-2014, for a total sample size of 177 former faculty members. The survey probed work history and experience, reasons for departure, and satisfaction at the SOM versus their current workplace. Statistical analyses included Pearson’s chi-square test of independence and independent sample *t*-tests to understand quantitative differences between clinical and non-clinical respondents, as well as coding of qualitative open-ended responses.

**Results:**

Eighty-eight faculty members responded (50%), including three who had since returned to the SOM. Overall, professional and advancement opportunities, salary concerns, and personal/family reasons were the three most cited factors for leaving. The average length of time at this SOM was shorter for faculty in clinical roles, who expressed lower workplace satisfaction and were more likely to perceive incongruence and inaccuracy in institutional expectations for their success than those in non-clinical roles. Clinical faculty respondents noted difficulty in balancing competing demands and navigating institutional expectations for advancement as reasons for leaving.

**Conclusions:**

AMCs may not be meeting faculty needs, especially those in clinical roles who balance multiple missions as clinicians, researchers, and educators. Institutions should address the challenges these faculty face in order to best recruit, retain, and advance faculty.

**Electronic supplementary material:**

The online version of this article (doi:10.1186/s12909-016-0830-y) contains supplementary material, which is available to authorized users.

## Background

Personnel turnover is an inevitable part of any organization’s life history. While turnover can signify the circulation of new ideas and perspectives in an academic community and across institutions, it also presents significant costs, especially if the reasons for turnover are tied to particular workplace challenges [1]. Faculty attrition from academic medical centers (AMCs) has become the object of discourse in recent years [2, 3]. In addition to costs associated with recruiting, hiring, and lost clinical income, the loss of intellectual capital, the foundation of an AMC, is another financial concern [4–6]. From a competitive standpoint, the loss of faculty members at one AMC may represent another institution’s gain. However, faculty departures from an AMC to the non-academic workforce could reflect serious issues with the academic culture and workplace more generally. Thus, faculty turnover can dampen workplace morale and climate, and exacerbate declining interest in pursuing or sustaining careers in academic medicine [7].

To promote interest and maintain excellence in academic medicine, we must understand what factors motivate faculty attrition from AMCs to different academic institutions as well other career paths. A survey of the literature suggests a wide array of factors can contribute to faculty departure, including: 1) dissatisfaction with institutional support, a lack of protected time for research, an unequal distribution of resources and rewards, an incongruence between described and actual work roles, and a lack of communication and support from departmental and institutional leadership [8, 9]; 2) perceptions that institutional and personal values are not aligned [10]; and 3) feelings of not belonging to or not being valued by the institution [11].

Against this backdrop, there is growing evidence that the factors influencing faculty departure depend also on the specific *structural* position that a faculty member occupies within an AMC [12]. As clinical and research-focused faculty roles have become more varied, so has the faculty experience and, conceivably, the propensity and reasons for leaving AMCs [13]. Research suggests that faculty in clinical roles are less likely to be at higher academic ranks, less likely to be satisfied with their progress towards academic promotion, and more likely to leave or express intent to leave academic medicine [14–17]. Academic physicians face challenges in juggling multiple responsibilities, navigating competing demands, and fulfilling expectations for promotion and advancement – pressures that could contribute to burnout and intent to leave [18]. In one study, faculty at an AMC who devoted more than 50% of their time to clinical care were significantly more likely than other faculty to report that tenure and promotion criteria were not reviewed at their annual evaluations, that they did not understand the criteria, and that they were dissatisfied with and less committed to academic medicine [16]. Clinical faculty may be most prone to feeling that their work is not valued, recognized, or rewarded by the institution, which may be in turn associated with intent to leave [15]. In addition, with tightening patient care budgets and the resulting pressure to increase their clinical workload, clinically focused faculty may find themselves lacking time for scholarly activities, which can disadvantage their career advancement and satisfaction [16].

In short, “why faculty leave” may depend on whether the individual’s role is primarily clinical or non-clinical. While discussions on this topic abound, in this study we sought to more systematically examine first the factors for faculty attrition and second, how factors for attrition differ across faculty roles. To do so, we surveyed faculty members who left one AMC about their experiences and satisfaction at the institution and their reasons for leaving. Our goals were two-fold: 1) to assemble a comprehensive picture of the faculty members’ experiences at the AMC and their reasons for leaving that AMC, using both quantitative and qualitative data; and 2) to understand whether this depended on the faculty role. The purpose of our study was to investigate why faculty left, whether reasons for leaving differed by clinical role, and what this means for efforts to advance faculty excellence in AMCs.

## Method

### Survey sampling

We designed and distributed an anonymous online survey in May and June 2010 to all 137 faculty members who left a research intensive United States west coast School of Medicine (SOM) between 1999 and 2009, and again in May and June 2015 to the 40 faculty members who left the SOM between 2010 and 2014 (Additional file 1). We chose these two time points in order to capture a sizable sample through 15 years of faculty departure data. The list of all these departing faculty members was obtained from the Office of Academic Affairs of the SOM. Respondents were emailed the survey. Eighty-eight of the invited participants responded to the survey (50% response rate; we compare the demographic profile of respondents to that of the entire population of departing faculty in the Results section below). Three respondents had left but subsequently returned to the SOM on different terms or under different faculty roles. For the purpose of this study, we focused our analysis on the 85 respondents who had permanently left the SOM.

### Survey design

We designed the survey to incorporate dimensions of faculty experience in addition to reasons for leaving. We drew from two established surveys: the faculty attrition survey at the University of Wisconsin-Madison, which guided questions on workplace satisfaction and reasons for leaving, and a survey used in one of the author’s published papers on faculty careers, which guided questions on support in the workplace [19, 20]. We included questions on time use, perceived accuracy of stated and expected roles, and perceptions of recognition and support. The survey ended with two open-ended questions inviting respondents to elaborate on their experiences at and reasons for leaving the SOM. We describe our main variables of interest below.

### Survey measures

The questionnaire was comprised of all the questions outlined herein. Given often-cited challenges in balancing roles and responsibilities, we asked respondents to cite the percentages allotted to clinical work, research, teaching, and administration in their appointment letters at the SOM. We asked, in their opinion, how closely their actual time use reflected these stated appointment percentages (*not closely, fairly closely,* or *closely*); if they had further comments on actual time use versus appointment percentages (open-ended); and whether they felt their contracts accurately reflected the institution’s expectations for their success (*1 = inaccurately* to *5 = accurately*). For respondents in clinical roles, we asked them to rate whether they felt the hospital gave them enough support to be successful in fulfilling criteria for their academic advancement, and how well they felt the hospital administration understood and supported their academic mission (*1 = not at all* to *5 = a lot*).

We next asked respondents to rate their satisfaction at the SOM and at their current workplaces on 18 items related to the workplace experience (*1 = very dissatisfied* to *5 = very satisfied*). Principal component analysis and varimax rotation techniques identified four factors – satisfaction with guidance, leadership, work environment, and institutional support – and Cronbach’s alpha coefficient of reliability indicated a high level of consistency among responses to the items in each factor, from 0.81 to 0.94 (see notes in Table 4 for the items in each factor). We calculated each factor as the mean of the factor’s composite items. Four items (flexibility, salary, opportunity for spouse, and work-life balance) did not load onto any factors and were analyzed separately.

To more directly understand specific reasons for leaving the SOM, we compiled a list of commonly cited factors for faculty attrition, drawn from literature [21] and the authors’ institutional experiences, and asked respondents whether these factors (listed in Table 5) were primary reasons for their departures from this SOM (*1 = yes, 0 = no*).

At the end of the survey, we asked respondents in an open-ended question to elaborate on the factors that affected their decisions to leave this SOM and to address any other issues not yet mentioned in the survey.

### Data analysis

We generated descriptive statistics summarizing the profiles of our respondents and compared responses by faculty roles in clinical and non-clinical spheres. We focus our analyses on those whose work encompasses all three components of the mission including clinical care, compared to those whose work focuses exclusively on the research and/or educational missions. Since the start of the study, an additional track has been added that focuses on clinical and educational work, with low emphasis on research. This track is not included in our analysis.

Pearson’s chi-square test of independence and two-tailed independent samples *t*-test were used to assess statistical significance in comparisons of categorical responses and comparison of means between clinical and non-clinical groups, respectively. Statistical analysis was performed using Stata version 12. Open-ended comments at the end of the survey were coded using both open coding to understand the overall factors affecting decisions to leave the SOM and focused coding to understand the relevant patterns of these factors across interviews.

## Results

### Respondent profile

Of the 85 respondents, 58 (68%) identified as male and 27 (32%) identified as female. Most identified as White (*n* = 68, 80%) or Asian (*n* = 8, 9%); others (*n* = 9, 11%) identified as Black (*n* = 3, 4%), Native Hawaiian or Pacific Islander (*n* = 1, 1%), Hispanic (*n* = 1, 1%), “Other” (*n* = 2, 2%), or two or more races (*n* = 2, 2%). The mean age of respondents was 45.0 years (Table 1).

**Table 1:** Profile of respondents in authors’ 2010-2015 survey of faculty (*N* = 85) who left SOM between 1999 and 2014

	*n* (%) or mean (standard deviation)
Gender	
Male	58 (68%)
Female	27 (32%)
Race/ethnicity^†^	
White	68 (80%)
Asian	8 (9%)
Black	3 (4%)
Native Hawaiian/Pacific Islander	1 (1%)
Hispanic	1 (1%)
Two or more races	2 (2%)
Other	2 (2%)
Age	45.0 (7.1)
Line at SOM*	
Clinical	47 (55%)
Non-Clinical	38 (45%)
Rank at SOM	
Assistant professor	43 (51%)
Associate professor	27 (32%)
Full professor	15 (17%)

^†^ *p* < .10 *p < .05 for Pearson’s chi-square test of independence respondents and non-respondents

Over half of the respondents (*n* = 47, 55%) were in a primarily clinical role at this SOM. At the time of their departures, 43 (51%) were assistant professors, 27 (32%) were associate professors, and 15 (17%) were full professors (Table 1). This profile resembles the faculty population distribution by role and the rank at the SOM and these sample demographics do not differ significantly from those of non-respondents: 66 male (*n* = 92, 72%); 58 White (63%); mean age 44.0 years; 66 (72%) clinical appointments; 52 (57%) assistant, 28 (30%) associate, and 12 (13%) full professors. 27 (33%) of respondents who left worked in surgical specialties and 6 (<1%) in basic science research. 55 (63%) of faculty who left the SOM specialized in non-surgical fields predominantly in Pediatrics and Medicine (34%).

Among all respondents, the average time spent at this SOM was 6.6 years, and after leaving the SOM, most went on to work in other academic institutions (*n* = 53, 62%; Table 2). There were significant differences between clinical and non-clinical respondents. For instance, the average time spent at this SOM was significantly lower among respondents in clinical roles (5.8 vs. 7.5 years, respectively; *p* < 0.05). An important similarity, however, was that among both clinical and non-clinical respondents, the majority remained in the academic setting (i.e., academic tenure or clinical/teaching non-tenure positions in another academic institution) (*n* = 25, 53% among clinical and *n* = 28, 74% among non-clinical) as opposed to moving to non-academic settings (i.e., health care organizations, private practice, or other).

**Table 2:** Comparisons by rank at SOM, years spent at SOM, and current employment setting among respondents in authors’ 2015 survey of faculty who left SOM between 1999 and 2014

	*n* (%) or mean (standard deviation)					
	All respondents		Clinical Line		Non-Clinical Line	
Rank at SOM						
Assistant professor	47 (55%)		30 (64%)		17 (36%)	
Associate professor	25 (30%)		12 (52%)		13 (48%)	
Full professor	13 (15%)		5 (38%)		8 (24%)	
Years at SOM^§^	6.6 (4.0)		5.8 (3.2)		7.5 (4.7)	
Current employment setting – academic vs. non-academic^†^						
Academic (academic tenure position or clinical/teaching non-tenure position in an academic institution)	53 (62%)		25 (53%)		28 (74%)	
Gender (% F in academic positions vs M)^a^	15(56%)	38 (66%)	8 (42%)	17 (61%)	7 (88%)	21 (70%)
Non-academic (health care organization, private practice, other)	32 (38%)		22 (47%)		10 (26%)	
Gender (% F in non-academic positions vs M)	12 (44%)	20 (34%)	11 (58%)	11 (39%)	1 (12%)	9 (30%)

^**§**^ *p* < .05 for independent samples t-test of difference in means between Clinical Line and Non-Clinical Line

^†^ *p* < .10 for Pearson’s chi-square test of independence between Clinical Line and Non-Clinical Line going to Academic vs. Non-Academic Medicine

^**a**^ No significance for Pearson’s chi-square test of independence between Female vs. Male in Academic vs. Non-Academic Medicine

We note that in this sample, similar percentages of men and women moved to other academic institutions (66% and 56%, respectively; differences are not statistically significant) compared to non-academic positions (34% and 44%, respectively; differences are not statistically significant; Table 2).

### Time use and perceptions of support

Respondents in clinical roles were significantly more likely to perceive that their actual time use did not closely reflect their appointment percentages (32% among clinical respondents vs. 17% among non-clinical respondents; *p* < 0.001; Table 3) and that their contracts inaccurately reflected the institution’s expectations about what it takes to be successful (40% in primarily clinical roles vs. 19% in primarily research roles; *p* < 0.01). Among clinical respondents, over half (*n* = 21, 58%) indicated “a little” or “not at all” when asked whether the hospital supported their success in fulfilling criteria for advancement, and a similar percentage (*n* = 20, 57%) indicated “not well” or “not well at all” when asked whether the hospital administration understood and supported their academic mission (Table 3).

**Table 3:** Respondents’ reported percentages of time use at SOM and perceptions of accuracy of contracts to institutional expectations for success, in authors’ 2015 survey of faculty who left SOM between 1999 and 2014

	*n* (%)			Valid *n*		
	All respondents	Clinical Line	Non-Clinical Line			
Actual time use reflected percentages in the appointment letter**				80	44	36
Not closely	20 (25%)	14 (32%)	6 (17%)			
Fairly closely	46 (57%)	27 (61%)	19 (53%)			
Closely	14 (18%)	3 (7%)	11 (30%)			
Contract reflected institution’s expectations about success**				69	38	31
Somewhat inaccurately-Inaccurately	21 (30%)	15 (40%)	6 (19%)			
Neutral	18 (26%)	13 (34%)	5 (16%)			
Somewhat accurately-Accurately	30 (44%)	10 (26%)	20 (65%)			
Hospital support in fulfilling criteria for advancement				46	36	10
A little-Not at all	N/A	21 (58%)	N/A			
Some	N/A	6 (17%)	N/A			
A lot-Quite a bit	N/A	9 (25%)	N/A			
Hospital administration understands and supports the academic mission				45	35	10
Not well-Not well at all	N/A	20 (57%)	N/A			
Fairly well	N/A	8 (23%)	N/A			
Well-Very Well	N/A	7 (20%)	N/A			

^†^ *p*< .10 * *p*< .05 ** *p*< .01 for Pearson’s chi-square test of independence between Clinical Line and Non-Clinical Line

Thirty-four respondents wrote in additional comments on their actual time use versus the appointment percentages. Comments in this section primarily described challenges in terms of lack of protected time for research and for those in clinical roles, more time spent in clinical work than expected. For example, these comments described:

“Most research was done on my own time, outside of the working hours.” (Clinical respondent)

“125% of time was spent on clinical [work].” (Clinical respondent)

“No real time for anything but clinical work.” (Clinical respondent)

These themes resurfaced in respondents’ open-ended comments that expanded on why they left the SOM (discussed in section below, “Why they left: insights from open-ended comments”).

### Satisfaction “then and now”

For the factors and items related to workplace satisfaction, we compared across faculty roles the percentage of respondents whose average satisfaction ratings were above 4 (i.e., “somewhat” to “very” satisfied). As expected, percentages expressing satisfaction were generally higher with respect to current workplaces than with respect to this SOM (Table 4). Compared to non-clinical respondents, faculty in clinical roles were somewhat less likely to express satisfaction with leadership and institutional support at the SOM (*p* < 0.10), and significantly less likely to express satisfaction with salary, flexibility, opportunity for partner/spouse, and work-life balance at the SOM (*p* < 0.05). Both sets of respondents were similar in their expressed satisfaction with their current workplaces.

**Table 4:** Percentage of respondents who were “somewhat” to “very” satisfied with various dimensions of SOM and current institutions, in authors’ 2015 survey of faculty who left SOM between 1999 and 2014

	*n* (%)					
	At SOM			At current workplace^**a**^		
	All respondents	Clinical Line	Non-Clinical Line	All respondents	Clinical Line	Non-Clinical Line
Factors						
Guidance	44 (54%)	20 (46%)	24 (65%)	66 (84%)	31 (74%)	35 (95%)
Leadership^†^	38 (48%)	17 (39%)	21 (58%)	50 (76%)	24 (66%)	26 (87%)
Environment	61 (74%)	32 (73%)	29 (76%)	75 (96%)	39 (95%)	35 (95%)
Institutional support^†^	41 (50%)	16 (36%)	25 (66%)	63 (82%)	31 (76%)	32 (89%)
Items						
Salary**	30 (37%)	10 (23%)	20 (53%)	69 (90%)	36 (86%)	33 (94%)
Flexibility*	42 (53%)	17 (39%)	25 (71%)	64 (81%)	33 (92%)	31 (84%)
Opportunity for partner/spouse*	21 (34%)	7 (22%)	14 (48%)	30 (59%)	15 (54%)	15 (65%)
Work-life balance*	34 (41%)	13 (30%)	21 (55%)	63 (81%)	34 (92%)	29 (83%)

^†^ *p* < .10 * *p* < .05 ** *p* < .01 for Pearson’s chi-square test of independence between Clinical Line and Non-Clinical Line “At SoM”

^**a**^Only one (salary) was significant among “At current workplace” group

*Note:* The factors contain the following items: 1) Guidance: satisfaction with orientation to the institution at time of hire, orientation to the department/division at time of hire, tenure and promotion mentoring, informal mentoring and guidance (alpha = 0.89); 2) Leadership: satisfaction with annual counseling with department chair/division chief, department chair/division chief (alpha = 0.89-0.94); 3) Environment: satisfaction with collegiality of faculty in the department/division, collegiality of faculty as a whole, treatment of you by others, connectedness to others within your department/division, connectedness to others outside your department/division (alpha = 0.88); 4) Institutional support: satisfaction with protected time for research, administrative support of your clinical work, consistency and clarity of promotion criteria (alpha = 0.81-0.86)

Valid N’s, All respondents – Clinical Line – Non-Clinical Line, are as follows:

1. Guidance: at SOM, 81 – 44 – 37; at current workplace, 79 – 42 – 37

2. Leadership: at SOM, 80 – 44 – 36; at current workplace, 66 – 36 – 30

3. Environment: at SOM, 82 – 44 – 38; at current workplace, 78 – 41 – 37

4. Institutional support: at SOM, 82 – 44 – 38; at current workplace, 77 – 41 – 36

5. Salary: at SOM, 81 – 43 – 38; at current workplace, 77 – 42 – 35

6. Flexibility: at SOM, 79 – 44 – 35; at current workplace, 79 – 36 – 37

7. Opportunity for partner/spouse: at SOM, 61 – 32 – 29; at current workplace, 51 – 28 – 23

8. Work-life balance: at SOM, 82 – 44 – 38; at current workplace, 78 – 37 – 35

### Factors that influenced decisions to leave

Respondents were given a list of factors commonly cited for leaving and asked to “check all that apply” as primary reasons for their exits from the west coast SOM; thus, respondents could check more than one factor as reasons for leaving. The top three factors were professional and advancement opportunities (*n* = 56, 66%), concerns about salary (*n* = 46, 54%), and personal/family reasons (*n* = 34, 40%) (Table 5). The clear front-runner among non-clinical respondents, professional and advancement opportunities (*n* = 31, 82%), was significantly less cited by those in clinical roles (*n* = 25, 53%; *p* < 0.01). Instead, the primary concern for those in clinical roles was salary (*n* = 28, 60%), which was less (though not statistically significantly) important among those in non-clinical roles (*n* = 18, 47%).

**Table 5:** Percentage of respondents who indicated the following factors were primary reasons for leaving SOM, in authors’ 2015 survey of faculty who left SOM between 1999 and 2014

	n (%)		
	All respondents (*n* = 85)	Clinical Line (*n* = 47)	Non-Clinical (*n* = 38)
Professional and/or advancement opportunities**	56 (66%)	25 (53%)	31 (82%)
Salary	46 (54%)	28 (60%)	18 (47%)
Personal/family reasons	34 (40%)	21 (45%)	13 (34%)
Issues with support (recognition, appreciation, etc.)	29 (34%)	17 (36%)	12 (35%)
Issues with research support	24 (28%)	16 (34%)	8 (21%)
Issues with clinical support**	13 (15%)	13 (28%)	0 (0%)
Geographic location	13 (15%)	8 (17%)	5 (13%)
Issues with diversity	3 (4%)	2 (4%)	1 (3%)
Discrimination	4 (5%)	3 (6%)	1 (3%)
Other	18 (21%)	8 (17%)	10 (26%)

^†^ *p* < .10 * *p* < .05 ** *p* < .01 for Pearson’s chi-square test of independence between Clinical Line and Non-Clinical Line

### Why they left: insights from open-ended comments

At the end of the survey, sixty-four respondents (75%) provided open-ended comments on their reasons for leaving the SOM. We used these responses to extract further insight into why these faculty members left the SOM. An important observation was that several respondents across both clinical and non-clinical roles described positive experiences at the SOM. They noted, for example:

“I have a very positive experience as a [non-clinical] faculty member at the SOM. I was well funded and had very good support from my department/division. The major reason I left was a unique opportunity to be part of a very innovative company.” (non-clinical respondent)

“My years at [the SOM] were the best years of my academic and personal life. The collaborative nature of the medical school/university and the accessibility of great minds and material is unmatched.” (clinical respondent)

“I loved [the SOM]. I was impressed by colleagues and staff. I felt everyone was working to help everyone advance and succeed.” (non-clinical respondent)

Concerns about salary (often associated with cost of living) and personal/family factors – two of the top three cited factors in Table 5 – were mentioned by a number of respondents across clinical and non-clinical roles as reasons for their departures (e.g., *“cost of living was a major part of my decision to leave;” “I predominantly left for family reasons;” “wanted to return home”*). The majority of comments, however, related reasons for leaving to specific experiences and challenges they encountered while pursuing their careers at the SOM. Many of these comments focused on professional and advancement opportunities – the top cited factor in Table 5. At the same time, comments also revealed nuances in how opportunity played into their decisions to leave. We discuss below the main themes (in bold) and representative quotes that emerged from these comments.

Departures from the SOM were often motivated mainly by **outside opportunities that respondents felt provided more room to develop their research interests** (e.g., *“the sole reason [for leaving the SOM] was for expanded [research] opportunities at [new organization]”*), more room to grow in leadership (e.g., *“opportunity to lead a division at another great medical school”*), or more career support (e.g., *“I was offered a fellowship, training, funding, and a more flexible schedule at [another] medical center”*). Similarly, respondents across both clinical and non-clinical roles described not only other opportunities but also **a perceived lack of opportunity at the home institution** as reasons for leaving the SOM. Among non-clinical respondents who commented on this issue, their comments described a lack of infrastructural support for their scientific work (e.g., *“lack of space/resources for developing laboratory”*) and a lack of opportunity for advancement to leadership positions (e.g., *“my potential for leadership was not fully embraced and therefore I took a superb opportunity elsewhere*.”) In addition to the perceived lack of opportunity and support, these comments suggested that respondents **did not feel valued or recognized**. As one respondent described:

“[At my current workplace] I feel validated and am able to perform at my peak. I felt that I was not really seen for what I could achieve [at the SOM].”

Similarly, among clinical respondents who discussed the lack of opportunity at the SOM, their comments described **a perceived lack of room for advancement** (e.g., *“I had no opportunity for further academic and administrative advancement”*) and a sense that they were not valued or recognized (e.g., *“I did not feel valued [at the SOM]”*). Particularly prevalent in their comments, however, were descriptions of specific challenges in balancing competing demands – challenges that were also noted in earlier comments on time use. Comments detailed the issue of balancing clinical work with research and teaching; they described, for example:

“Each year we were asked to see more and more patients… The stress level was astronomical… [Because of these conditions] there was no time or energy left for the only reasons to stay [at the SOM] – to teach or do research.”

“I left because I was over-scheduled clinically for perennial faculty shortages. My concerns were not appreciated by department leadership and I felt I needed to move on in order to develop other academic interests. I felt then that the demands for promotion were not possible given the taxing clinical demands and I saw no way out.”

The difficulty in balancing competing demands and finding time for non-clinical activities among clinical faculty has been described in literature, and respondents in this study were not immune to it [7]. In addition to these qualitative comments, Table 5 showed that over a quarter of clinical respondents indicated “lack of clinical support” to be a reason for their departures from the SOM. Furthermore, like clinical faculty in other studies, clinical respondents here described feeling undervalued given the traditional emphasis on research in the rewards system, noting for example that *“the clinician-educator role is grossly undervalued,”* and a desire to find an environment with greater *“acknowledgement of clinical care”* [22, 23]. Some respondents also described feeling a lack of general support for clinical researchers; as one respondent commented, *“clinical researchers were not provided with departmental or school support for their clinical programs, and thus are often swamped by clinical responsibilities.”*

These factors have implications on opportunities for advancement for clinical faculty members. As one respondent explained, *“The major challenge was balancing a heavy clinical load with research activities, especially during the critical transition from junior level to more senior level positions.”* Another respondent noted that he had to leave the SOM because he was *“clinically productive but not publishing.”* A third respondent described:

“I did not see opportunity for professional advancement in my clinical group, and I certainly did not have adequate time or resources to become an independent scientific investigator… I wanted to be in charge of something, be recognized, and be paid a fair salary for my level of expertise.”

The above comment revealed not only difficulties in balancing demands and gaining recognition, but also **difficulties in navigating institutional expectations for academic success**. In addition to conflicts between clinical demands and research requirements for advancement, guidelines for success for clinical faculty can often seem unclear or even unattainable [23, 24]. One respondent wrote that she had inadequate time to get outside grants and constantly felt behind, but “*no one could actually tell [her] whether [her] performance was adequate.”* Another respondent emphasized this issue and its implications on promotion:

“I was [in a primarily clinical role] – so the expectation was mainly [to generate clinical revenue] but be required to publish/research in order to be promoted – it is the [clinical] academic paradox. [the SOM is a] great school with some truly outstanding clinicians… But there were no clear guidelines that distinguished what “success” looks like. There was a very archaic rewards/promotion system – particularly for those [in clinical roles] who were expected to be as academically productive as [researchers].”

## Discussion

The vitality of an academic medical center depends on its faculty’s success. Understanding the reasons for faculty attrition can inform the challenges that faculty face in the academic workplace, as well as the efforts necessary to best support and advance faculty. In light of increasing variation in faculty roles within AMCs, it is furthermore important to investigate how the faculty experience and reasons for leaving may differ for faculty on different tracks. Our study’s findings, based on data from 85 faculty members who left one AMC, suggest that the reasons for faculty departure are multifaceted and tied to the faculty role occupied. Respondents in primarily clinical roles were significantly more likely than others to perceive incongruence and inaccuracy in institutional expectations for their work roles and for their career success. They expressed generally lower ratings of satisfaction with department leadership, institutional support, and workplace factors such as salary, flexibility, and work-life balance. While professional and advancement opportunities, salary, and personal/family reasons were cited by many respondents as reasons for leaving, those in a clinical role specifically described challenges in balancing competing demands and navigating institutional expectations for success. Length of stay at the SOM prior to departure was also significantly shorter among clinical respondents, suggesting that faculty attrition and turnover may be especially problematic among clinical faculty. However, it can also be argued that during a time of cutbacks in research funding, PhD faculty face greater challenges to career satisfaction and advancement than MDs since the latter can earn a living by practicing medicine.

This paper served to identify the main factors affecting faculty departure and to gain an initial understanding of how these might differ for those in clinical versus non-clinical roles. Future research into this area would do well to take the factors identified here to perform multivariate analyses, controlling for various demographic, compensation, and/or role characteristics.

Additionally, future work could examine cross-institutional contexts. A potential limitation with single-institution studies is that results may not be generalizable to the broader context or to other institutions that have a different mission focus or organizational structure. Despite being based on one institution, our findings comport with the widespread discussion about the changing landscape of academic medicine and the emerging challenges to faculty success. As AMCs contend with rising health care costs, changes in health care delivery and reimbursement, and tightening of patient care budgets, faculty grapple with decreasing research funding and increasing and competing time demands at work [7, 25–27]. Clinical faculty may be particularly vulnerable to pressures and stressors not only as AMCs become more dependent on clinical revenue, but also because promotion structures within AMCs are based on research and teaching, missions in which clinical faculty may not have time or resources to fully participate [22, 23, 28]. One study at another AMC found that the odds of attaining a higher academic rank are 85% lower for clinician researchers and 69% lower for clinician educators compared to research faculty [13]. Adding to extant literature on the challenges clinical faculty face with respect to promotion, institutional recognition and support, and burnout, our study relates these challenges to actual reasons for leaving an AMC. The results pose the bigger structural question of the viability of tracks like the MCL (clinical-research-education track) that affects all AMCs. Is the expectation of clinical productivity and competitive grant funding sustainable in the future, especially given the next generation’s expectations regarding work hours, a physician and hence clinical faculty shortage, and increasing competition for shrinking funds and discretionary time?

Interestingly, we found that similarly high percentages of women and men left the SOM for other AMCs, suggesting that women are as likely as men to remain in academic medicine. This resonates with recent research that found no gender difference in the intent to leave academic medicine [1]. However, significant gender gaps persist in women’s representation in senior and leadership positions, and there is much evidence that women face particular challenges in their career development, for example in finding mentors, facing hidden or unconscious bias, and encountering situations in the work environment that elicit stereotype threat [29–32]. That women faculty may face additional barriers, but at the same time may be no more likely to leave academic medicine for other settings, confers all the more import to understanding the factors that may impede women’s academic careers.

Given that many faculty remain committed to working in academic medicine, it is imperative that AMCs understand the specific needs of clinical and non-clinical faculty and develop organizational structures to best support and advance them. Expansion beyond single-site studies to multi-site studies can enable larger samples and cross-institutional analyses. While in this study the numbers were too small to separate non-clinical faculty who were not in tenure tracks, with larger samples we can begin to understand similarities and differences at finer gradations of multiple kinds of faculty tracks (e.g., tenure-track researcher vs. non-tenure-track researcher). Examinations of policies and practices across institutions can further identify ways to effectively retain and facilitate faculty success.

The challenge is for AMCs to define, structure, and align promotion and rewards system with the roles, values, and needs of faculty on different tracks. With growing numbers of faculty in primarily clinical roles, it is all the more important that institutional policies fit the interests and needs of these faculty members [33]. It is also clear that organizational support for faculty members’ academic pursuits, whether they be clinical, research, teaching, or administration focused, is critical to faculty satisfaction and success in an institution and beyond. In addition to clarifying promotion and rewards processes and providing material support, our findings suggest that ensuring, that faculty feel valued and recognized is central to faculty development and retention. Innovative strategies are needed to facilitate a culture in which faculty across all roles experience less burnout, more flexibility in their work roles, and a greater sense of satisfaction and recognition of their achievements and success.

## Conclusions

In conclusion, our systematic examination of why faculty leave an AMC, which considered the faculty’s specific position and experiences within the institution, provides much insight into stressors particular to various faculty roles and potential institutional strategies to address these challenges and can help faculty thrive. In light of arising challenges in academic medicine in the 21^st^ century, continued research on this front will be critical to understanding what AMCs must do to promote faculty and excellence in medical care, education, and research.

## Additional file

Additional file 1: Questionnaire (DOCX 33 kb)

## Abbreviations

AMC: Academic Medical Center
SOM: School of Medicine
