# Supplementary material for: Reasons for faculty departures from an academic medical center: a survey and comparison across faculty lines
Source: BMC Med Educ. 2017 Jan 10;17:8. doi: 10.1186/s12909-016-0830-y (PMC5223325; doi:10.1186/s12909-016-0830-y)
Supplement: Additional file 1: — Questionnaire (DOCX 33 kb) [file 12909_2016_830_MOESM1_ESM.docx]

| **FACULTY RETENTION AND ATTRITION PROJECT**  **SURVEY** | |
| --- | --- |
| **PART I. DEMOGRAPHIC BACKGROUND** | |
| 1. Year of birth | (Fill in) |
| 1. Years of age | (Fill in) |
| 1. Gender | (Choose one)  Male  Female |
| 1. Race/ethnicity | (May choose more than one)  White, Non-Hispanic  Black/African American, Non-Hispanic  American Indian/Alaskan Native  Asian/Asian American  Native Hawaiian/Pacific Islander  Mexican/Mexican American  Puerto Rican  Other Hispanic/Latino  Other – please specify (fill in) |
| 1. Household status | (Choose one)  Spouse/partner  In a serious relationship  Separated, divorced or widowed  Single |
| 1. Parental status | (Choose one)  Do not have children  Have children – (drop-down: number of children) |
| 1. Years of age of each child   *(Only for those with children)* | (Fill in: up to 5 children) |
| 1. Country of birth | (Choose one)  U.S.  Not U.S. – please specify country (fill in) |
| **PART II. EDUCATION AND WORK HISTORY** | |
| 1. What educational degrees have you earned? | (Choose as applicable)  M.D.  M.D./Ph.D.  Ph.D.  D.O.  Other (please specify) |
| 1. In what year did you receive your last degree? | (Fill in) |
| 1. At what institution did you receive your last degree? | (Fill in) |
| 1. At what institution did you receive your first job after your last degree? | (Fill in) |
| 1. Was your first job a post-doctoral fellow position? | No  Yes – please select type (drop-down: research / clinical) |
| 1. How many post-doctoral positions did you hold between your last degree and your first appointment as an assistant professor? | (Fill in) |
| 1. What academic track are you in? | (Choose one)  Clinician Instructor  MCL  UTL  NTL research  NTL teaching |
| 1. What is your current academic title? | Assistant Professor  Associate Professor  Full Professor |
| 1. In what year in your term are you? | (Fill in) |
| 1. Did you extend your term appointment? | No  Yes (drop down – years 1,2,3,more) |
| 1. Are you currently tenured?   *(UTL only)* | (Choose one)  No  Yes |
| 1. In what year did you receive your tenure?   *(UTL tenured only)* | (Fill in) |
| 1. Do you expect to receive tenure at your current institution?   *(UTL non-tenured only)* | (Choose one)  No  Yes |
| 1. In how many years will it be your tenure decision?   *(UTL non-tenured only)* | (Fill in) |
| 1. In what year did you start at your current institution? | (Fill in) |
| 1. How many years have you been at your current institution? | (Drop-down: number of years, including “less than a year” and “more than 10 years” options) |
| 1. For how many years were you employed at your last institution?   *(If length at the current institution is 4 or fewer years)* | (Drop-down: number of years, including “less than a year” and “more than 10 years” options) |
| 1. What are the percentages allotted to each of the following activities as listed in your most recent appointment letter? | (Fill in for each or drop-down: % categories)  Research  Clinical  Teaching  Administration |
| 1. Please provide your work history (appointment, institution, years). | (Fill in) |
| **PART III. WORKPLACE SATISFACTION AND EXPERIENCE** | |
| 1. Rate your satisfaction with the following at your current institution.  - Orientation to the institution at time of hire - Orientation to the department at time of hire - Tenure and promotion mentoring - Annual counseling with chair/chief - Department chair/division chief - Collegiality of faculty in the department/division - Collegiality of faculty as a whole (i.e., including outside the department/division) - Treatment of you by others (i.e., chair/chief, peer colleagues) - Connectedness to others within your department/division - Connectedness to others outside your department/division - Informal mentoring and guidance - Support of your research interests - Support of your clinical endeavors - Resources to perform your job - Flexibility - Salary and benefits - Opportunities for spouses/partners - Balance between work and home | (Choose one)  Very dissatisfied  Dissatisfied  Somewhat dissatisfied  Neither satisfied nor dissatisfied  Somewhat satisfied  Satisfied  Very satisfied |
| 1. Rate your satisfaction with the following at your current institution.  - Orientation to the institution at time of hire - Orientation to the department at time of hire - Tenure and promotion mentoring - Annual counseling with chair/chief - Department chair/division chief - Collegiality of faculty in the department/division - Collegiality of faculty as a whole (i.e., including outside the department/division) - Treatment of you by others (i.e., chair/chief, peer colleagues) - Connectedness to others within your department/division - Connectedness to others outside your department/division - Informal mentoring and guidance - Support of your research interests - Support of your clinical endeavors - Resources to perform your job - Flexibility - Salary and benefits - Opportunities for spouses/partners - Balance between work and home | (Choose one)  Very dissatisfied  Dissatisfied  Somewhat dissatisfied  Neither satisfied nor dissatisfied  Somewhat satisfied  Satisfied  Very satisfied |
| 1. What is the primary reason for your exit from your last institution? | (Choose as many as applicable)  Personal/family  Geographic location  Better professional opportunity  Advancement or promotion  Higher salary  Salary inequity  Lack of opportunities  Lack of advancement or promotion  Lack of critical resources (e.g., space, financial support, time)  Lack of support (e.g., recognition, appreciation)  Discrimination  Was asked to leave  Other – please specify (fill in) |
| 1. In your opinion, were any of the characteristics below a factor in your decision to leave your last institution?  - Age - Gender - Sexual orientation - Relationship status - Family status/responsibilities - Race/ethnicity - Disability - Religion - Inequality – specify - Other – specify | (Choose one)  No  Yes – (fill in if the “inequality” and “other” options are chosen) |
| 1. Did any of the below encourage you to leave your last institution?  - Department chair/division chief - Peer colleagues | (Choose one)  No  Yes |
| 1. Have you been recruited by an outside entity? | (Choose one)  No  Yes – please specify (fill in) |
| 1. Did you consult with anyone about your decision to leave? | (Choose one)  No  Yes |
| 1. Would you recommend your last institution to others? | (Choose one)  No  Yes |
| 1. What is the primary reason for coming to Stanford? | (Choose as many as applicable)  Personal/family  Geographic location  Better professional opportunity  Advancement or promotion  Higher salary  Salary inequity  Lack of opportunities  Lack of advancement or promotion  Lack of critical resources (e.g., space, financial support, time)  Lack of support (e.g., recognition, appreciation)  Discrimination  Was asked to leave  Other – please specify (fill in) |
| **PART IV. SUCCESS STRATEGIES AND NETWORK CHARACTERISTICS** | |
| 1. In your opinion, which of the following most determines success in your work? | (Choose one)  Research accomplishments  Clinical care  Both research and clinical care  Collegial relationships  All of the above |
| 1. Do you often feel isolated from  - Colleagues in your department/division? - Colleagues outside your department/division? | (Choose one)  Never  Rarely  Sometimes  Often  Very often |
| 1. Do you agree or disagree with the following?  - I normally express agreement with a senior colleague’s viewpoint, even if I do not completely share his/her opinion. - As a general rule, I keep my family and social life out of my conversations with other faculty. - My performance at my current institution has been evaluated and rewarded fairly. - In this department/division, there is a norm that a high level of competition is good for productivity. - Faculty and students of the same gender work better together. | (Choose one)  Strongly disagree  Disagree  Somewhat disagree  Neither agree nor disagree  Somewhat agree  Agree  Strongly agree |
| 1. How often do you  - Talk to peers about happenings in the department? - Ask peers for advice and support? - Socialize with peers *in* the workplace in a typical week? - Socialize with peers *outside* the workplace in a typical week? | (Choose one)  Never  Rarely  Sometimes  Often  Very often |
| 1. Do people come to you for work-related advice and information? | (Choose one)  No  Yes |
| 1. Would you agree if people ask you to volunteer to help with an activity that is of no benefit to you? | (Choose one)  No  Yes |
| 1. How important are colleagues to your personal feeling of well-being? | (Choose one)  Not important at all  Unimportant  Somewhat unimportant  Neither important nor unimportant  Somewhat important  Important  Very important |
| 1. Do you have a support structure within the school if you have a professional problem? | (Choose one)  No  Yes |
| 1. Do you have a role model  - In your department/division? - Outside your department/division? | (Choose one)  No  Yes |
| 1. Do you serve as a peer mentor or advisor for colleagues? | (Choose one)  No  Yes |
| 1. Think about a professional decision that you have faced in the past. Do you have a network for support and advice  - Within your department/division? - Within the school at large? - Outside the school? | (Choose one)  No  Yes |
| 1. Has your number of personal contacts for professional advice or social support grown over the past two years? | (Choose one)  Shrunk  Stayed the same  Grew |
| 1. Compared to others at the same stage in their careers as you are, do you feel more or less connected than your peers? | (Choose one)  Much less connected  Less connected  About the same  More connected  Much more connected |
